# An Improved HMM-Based Approach for Planning Individual Routes Using Crowd Sourcing Spatiotemporal Data

**DOI:** 10.3390/s20236938

**Published:** 2020-12-04

**Authors:** Tao Wu, Zhixuan Zeng, Jianxin Qin, Longgang Xiang, Yiliang Wan

**Affiliations:** 1 Hunan Key Laboratory of Geospatial Big Data Mining and Application, Hunan Normal University, Changsha 410081, China; blackender@hunnu.edu.cn (T.W.); Zhixuan@smail.hunnu.edu.cn (Z.Z.); wanylir@hunnu.edu.cn (Y.W.); 2 College of Resources and Environmental Sciences, Hunan Normal University, Changsha 410081, China; 3 State Key Laboratory of LIESMARS, Wuhan University, Wuhan 430079, China; geoxlg@whu.edu.cn

**Keywords:** hidden Markov model, route planning, crowd sourcing spatiotemporal data

## Abstract

With the rapid development of LBSs (location-based services) in recent years, researchers have increasingly taken an interest in trying to make travel routes more practicable and individualized. Despite the fact that many studies have been conducted on routes using LBS data, the specific routes are deficient in dynamic scalability and the correlations between environmental constraints and personal choices have not been investigated. This paper proposes an improved HMM-based (hidden Markov model) method for planning personalized routes with crowd sourcing spatiotemporal data. It tries to integrate the dynamic public preferences, the individual interests and the physical road network space in the same spatiotemporal framework, ensuring that reasonable routes will be generated. A novel dual-layer mapping structure has been proposed to bridge the gap from brief individual preferences to specific entries of POIs (points-of-interest) inside realistic road networks. A case study on Changsha city has proven that the proposed method can not only flexibly plan people’s travel routes under different spatiotemporal backgrounds but also is close to people’s natural selection by the perception of the group.

## 1. Introduction

Along with the swift development and evolution of emerging services, including internet cloud computing, Internet of Things and social networking services, and the increasing use of mobile devices, LBSs (location-based services) are booming. It is the outbreak of LBS that promoted studies and applications of intelligent transportation, in which various intelligent terminals, especially mobile intelligent devices with location positioning, played a sensor-like role, contributing more and more crowd sourcing spatiotemporal data. Route planning, as one of the hot issues in the field of artificial intelligence, has attracted more and more attention from researchers. The research focus in related areas has shifted from straightforward POI (point-of-interest) recommendations to more complex personal route planning. The route planning of individual travel is more meaningful only when the dynamic spaiotemporal information, public preferences and personal choices are considered comprehensively. For example, when people travel to a strange city, they not only need to know and choose specific POIs they are interested in, but also need to consider the routes to complete their visits to these POIs according to the traffic situation of the city. Choosing the optimal route is complicated because it depends on many factors. Therefore, if he does not use route optimization, he stands to lose time, money and travel activities from lost efficiency. The advantage of route optimization after incorporating these considerations is that it can satisfy people’s travel activities and be close to people’s natural choices, and thus is popular with the public.

Route planning is about how to create driving directions or routes for multiple locations in the most efficient way. Generally, there are three important components to planning a route. They are the elements, a process and results. In other words, they can be expressed as addresses or stops, constraint conditions and individual intentions. Under a constraint condition, the route formed by combining road network and POIs can satisfy people. In order to achieve this, they need to constantly optimize these constraints in the new path planning. In particular, the needs of individuals or the public and their dynamic preferences for POIs are considered to determine the choice of POIs. Then, they need to plan visits to these POIs as a connected itinerary for designing a personalized path plan. This kind of personalized travel route can be close to people’s natural choices and conform to the public’s ideas and preferences. At the same time, in combination with personal constraints, the most suitable route taking the least time can be made according to the actual road network. Therefore, it needs to take the public’s preferences for different locations, personal intentions and spatial information of the road network into the route planning considerations.

In previous studies, many achievements have been reached in successive POI recommendations and tourism route planning. It has fascinated scholars, starting with existing traditional approaches [1,2], and expressly proposing novel optimal route planning methods for moving objects [3,4]. However, important issues are still open. These previous studies focused on managing sequences of POIs or road segments, rather than applying dynamic attitudes to various relevant elements. It is a dynamic problem; in different time and location conditions, the appropriate trip is different from the content of the route. When people are arranging travel plans, the non-static factor is particularly important. If people go to visit a spot at an inappropriate time, they may arrive at the spot and not be able to visit it, which not only wastes time but also increases costs. The originally planned travel route has changed. A person will feel dissatisfied with this inappropriate route, which will affect the tourist’s mood.

Other important concerns are personalized needs and interests, which cannot be sufficiently combined for integration into the relationship between actual road network information and POIs. Facing these problems, the path uncertainty which is caused by various factors should be reduced. Good route planning takes into account all the essential details (restrictions, ferries, routing optimizing and so on). For example, take the PersTour algorithm proposed by Lim et al. [5]. The algorithm recommends tour itineraries with POIs and visit durations tailored to the interest preferences of individual tourists. This personalization is based on both POIs’ popularity and time-based user interests. To a certain extent, this algorithm can solve the problem of the correlation between the personalized demand of route planning and the popularity of POIs. However, the algorithm ignores the spatiotemporal dynamics of POIs, and it ignores the changes in the popularity of POIs in different periods. There are also some traditional methods that often need to collect large amounts of data, which results in a longer implementation cycle. For a more comprehensive solution, it is necessary to consider the public’s preference for POIs in different periods in order to obtain the popularity of POIs in different periods. By combining this factor with the previous factors to design personalized route planning, the final recommended route will be more excellent and the implementation cycle will be shortened.

In addition, the actual road network information is also a core factor in route planning. As the main component of urban space, the road is the carrier of actual travel routes in the urban environment. The topological structure of a road network directly affects the specific route. At the same time, the spatial relationship between the road network and POI is also an influencing factor. The pattern of an urban road network is often closely related to the distribution of POIs. Therefore, the actual road network information cannot be ignored. The combination of POIs and the road network is helpful for the study of route planning.

In this paper, an improved HMM (hidden Markov model) is proposed, which can make dynamic route planning according to the personal willingness, heat of POIs and path constraints. The improved HMM model in this paper maps the dynamic public preference information in open source data to the physical road network space. The public’s preference for different locations, individual intentions and actual road network spatial information are taken into account. The public’s preference for POI is fully reflected in the actual path. We designed the HMM structure of a dual-layer mapping, and described the double random process composed of the hidden state represented by POI–POI and the observation state sequence based on the user selection. We are able to extract the route network data and POI data, and then POIs are classified by their attributes (parks, universities, shopping, etc.) and the heat information of a single POI in each month and quarter. The method to generate the required parameters was analyzed within HMM. Finally, the proposed method was evaluated on the real data and compared with traditional route planning methods. The experimental results show the effectiveness and feasibility of this model in routing planning tasks.

To summarize, the major contributions of this paper are:

- It presents an improved dual-layer HMM structure. The improved dual-layer mapping HMM can reduce the spatial uncertainty interference caused by traditional methods on POI and road network data. The new method of personal route planning is proposed based on HMM, which can make dynamic route planning according to the personal willingness, heat of POI and path constraints.
- It proposes the method for planning personalized routes with POIs and visit sequence based on the dynamic public preferences, individual interests and the physical road network space. The method maps the dynamic public preference information in crowd sourcing spatiotemporal data to the physical road network space. The public’s preference for different locations, individual intentions and actual road network spatial information are taken into account. It tries to find common ground and balance between public big data information and personal tendency.
- This method also combines spatiotemporal features with static data. It emphasizes the spatiotemporal dynamics, that is, the interaction between the POIs and environment in different periods. It is more conducive to our analysis.
- The method may be useful not only for route planning, but also for selecting destinations. The resulting route is closer to people’s natural selection.

The structure of this paper is organized as follows: Section 2 briefly describes related research. Section 4 introduces the dataset, and the method of personal route planning is proposed based on the hidden Markov model. Section 5 shows the experimental results and related analysis. Conclusions and future work are provided in Section 6.

## 2. Related Work

This section first briefly introduces the latest works on route planning. The route planning methods have been widely studied. Among these studies, researchers recommend POIs and routes through different research methods and algorithms. The research related to POI recommendations and route planning problems is mainly summarized.

Based on the research of route recommendation, Cheng et al. [6] proposed a novel matrix factorization method which is embedded in FPMC-LR (the personalized Markov chains and the localized regions) based on LBSNs (location based social networks); it generated successive POIs recommendations in the form of individualized Markov chains. By considering the influence of time, Zhao et al. [7] proposed a STELLAR (spatial-temporal latent ranking) method for modeling a user’s most recent check-in and the historical querying activities. Gau et al. [8] and Lu et al. [9] proposed a new method to make successive POI recommendations, named UGSE-LR. To achieve successive POI recommendations, Lin et al. [10] integrated users’ preferences, the influence of time and the influence of geography, and used the MF to analyze the interaction among them. Huang et al. [4] proposed a new framework named DRPS (dynamic recommendation of POI sequence), which is a dynamic recommendation framework for POI sequences. Ying et al. [11] proposed a MEAP-T (time-aware metric embedding approach with asymmetric projection) method to solve the asymmetry of POI transitions for recommending the most possible successive POIs. There are other studies on successive POI recommendation [12,13,14].

Researchers also use different data for POIs and route planning. Historical trajectory data are frequently used for route recommendations and route predictions, such as effective path and GPS trajectories. Zhou et al. [15] used effective path coverage, which was determined by the associations among the POIs, and proposed a top-k POI recommendation model based on these data for personalized POI recommendation. Based on the historical trajectories, Chen et al. [16] developed a coherence expanding algorithm to establish the transfer network model, and a maximum probability product algorithm was proposed for route recommendation. A method for integrating heterogeneous tourism data was proposed by Bin et al. [17]. Then, they proposed an improved algorithm to obtain all kinds of candidate personalized routes. Majid et al. [18] and Cai et al. [19] proposed an itinerary system from a collection of geo-tagged photos to recommend routes. Lim et al. [5] exploited geotagged photographs to extract travel histories of users and proposed an algorithm named PersTour to recommend personalized routes. Zhao et al. [20] explored the sentimental spatial context and sentimental attributes of locations; a SPR (sentimental-spatial POI recommendation) method was proposed for mining the POIs; and a SPR model was used to plan a personalized recommendation. Aliannejadi et al. [21] and Fogli et al. [22] used Context-Aware to make a personalized POI and itinerary recommendations. Du et al. [23] utilized the travel text data to construct the travel route dataset, and proposed a novel travel path mining method which considered the features of these dataset to excavate the travel route. A new method which was used to mine the multi-source tourism information and integrate it was proposed by Bin et al. [24] to obtain visit sequences of POIs. Then, they proposed an algorithm for mining the sequence to generate candidate travel routes. Sansonetti [25] proposed a personalized recommender system by using LOD (linked open data). Devarajan et al. [26] and Huang et al. [27] used social cyber-physical systems for POI recommendations. Moreover, there are some algorithms for POI and route recommendations [28,29,30]. Ding et al. [31] and zhang et al. [32] used an improved neural network for POI recommendation. Based on naïve Bayes interest data mining machine learning, a smart tour route planning algorithm was proposed by Zhou et al. [1]. In addition, there are other studies on POI and route recommendations [33,34,35,36,37,38,39].

Many research studies used Markov chains which could capture the sequence pattern to predict or recommend routes and destinations. Newson et al. [40] used a HMM to detect the most likely road route. Based on the HMM, Mathew et al. [41] proposed a hybrid method which could cluster location histories to predict human mobility, and Qiao et al. [42] presented a HMTP (hidden Markov model-based trajectory prediction) algorithm to predict successive routes of moving objects. Lassoued et al. [43] proposed a novel algorithm based on the formalism of HMM for route and destination prediction. An algorithm based on a multi-order Markov model was proposed by Liu et al. [44] for recommending the POIs, which could predict the next POIs of users’ favorites. Combining the Markov model and PPM (prediction by partial matching) technique, Neto et al. [45] presented a novel predictor which could automatically predict the route and destination in real time.

It can be seen from these works that researchers recommend POIs or routes by designing different new methods and new models. However, these works recommend POIs directly, without considering the category of the POIs. The spatiotemporal dynamics of POI are rarely emphasized. Few studies’ recommended routes are based on the sequence of POIs selected by the user, and few studies considered the spatial-temporal correlativity to plan a route recommendation. Most of the related works have focused on route prediction or recommendation based on the past routes data. It is common to ignore the updates to the actual road network and fail to consider the impact of the interaction between a POI and the environment at different times during one’s travel. In order to solve the above problems, an improved HMM route planning method is proposed. This method uses crowd sourcing spatiotemporal data to plan personal routes. Crowd sourcing spatiotemporal data are used to extract dynamic public preferences, of which the projection is given consideration through corresponding environmental data. Reasonable routes are subsequently generated by synthesizing public preferences, individual intentions and specific environmental factors in this method.

## 3. Problem Statement

The problem of individual route planning in this paper is defined as:

Given a sequence of POIs of interest to the users, a road network and POI sets, obtain the optimal POI set and arrange it with optimal routes. This involves two key issues:

### 3.1. Subjects to POIs

POIs with the same attributes are treated as the same subjects. Categories of subjects are determined to facilitate user selection and to build the order of user access. The route planning task can be defined as follows: A set of user-selected subjects $S=(s_{1},s_{2},s_{3},...,s_{i})$, where $s_{i}$ is a specific subject including all POIs, denoted as $s_{i}=\left(p_{i1},p_{i2},p_{i3},\cdots ,p_{ij}\right)$. When the user completes the selection of subjects and determines the order of users access, the corresponding set of subjects should be associated with several sequences of optimal POIs from their parent subject sets. The biggest difficulty is how to plan a feasible route based on the actual spatial information.

### 3.2. POIs to projections

The location of a POI will not appear on the road. We have to calculate the projected position on the road segment corresponding to each point $p_{ij}$ [46]. If there is more than one road segment around the POI, it is possible to get multiple projected points. Define the projected point as $p_{ijk}^{\prime }$, where *i* represents the sequence number of a subject, *j* represents the sequence number of POI within the subject and *k* represents the sequence number of the projected point of the POI. The point of projection $proj(p_{ij},r_{j})$ is calculated by the Euclidean distance from the point to the line. Each POI contains one or a few projected points on the road network $p_{ij}=\left(p_{ij1}^{\prime },p_{ij2}^{\prime },\cdots ,p_{ijk}^{\prime }\right)$, which are the candidate points. The projection $proj(p_{ij},r_{j})$ of point $p_{ij}$ onto $r_{j}$:

(1) $$proj\left(p_{ij},r_{j}\right)=\underset{p_{ijk}^{\prime }\in r_{j}}{\arg\min ed\left(p_{ij},\;p_{ijk}^{\prime }\right)}$$

where $ed(p_{ij},\;p_{ijk}^{\prime })$ are the Euclidean distances between a POI and its projected points on the adjacent road segments.

**Figure 1:**
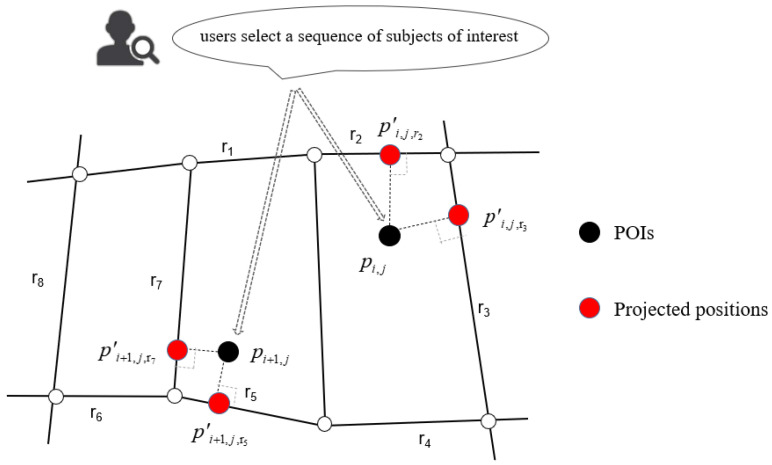
Projected positions of POIs $p_{ij}$ and $p_{i+1,j}$ on adjacent road segments.

Take the distance factor from the POIs to the actual road segments into consideration. It is not possible to project the POIs to all road segments. Therefore, a distance threshold will be set. Calculate all projected points from POIs to the neighboring road segments within the distance threshold. Figure 1, it explains the calculation of the projected points of the POI on the road sections.

## 4. Methodology

In this section, it is divided into several parts to describe. The content mainly includes: introducing the acquisition of data, including the related datasets, data characteristics and data sources. Then, we briefly describes the preprocessing of various data types. The improved HMM proposed in this paper is described in detail.

### 4.1. Framework

**Figure 2:**
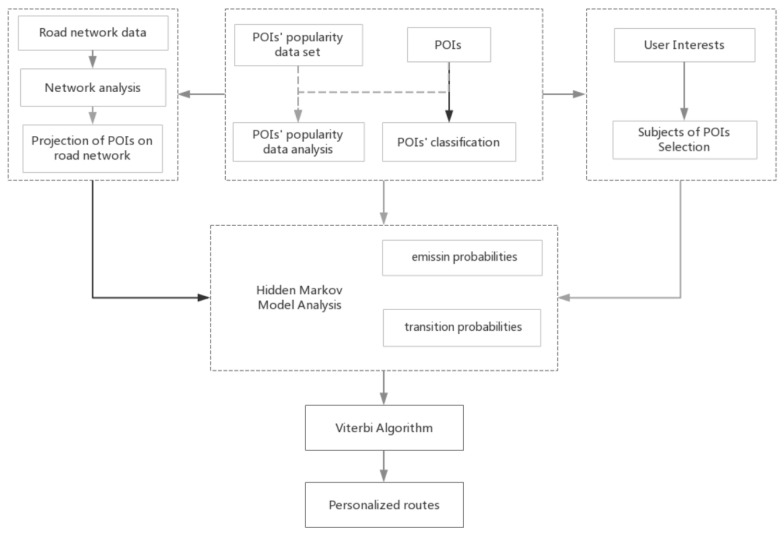
Flowchart of the route planning framework.

Figure 2 illustrates the architecture of the proposed method. First of all, the POI data and the heat data of POIs have to be integrated and preprocessed. By analyzing the popularity data of POIs, it can get most users’ preferences for POIs in different periods. By combining the actual road network data, the POIs will be mapped to the corresponding actual road segments to get the projections of POIs on the road segments. The Euclidean distance from the POI to the projection is calculated. The projection procedure is also included in the emission probability. Then, its calculates the great circle distance and route distance between POIs. According to the types of POIs selected by the user’s personal interests, a set of visit sequences is generated to obtain the user’s behavior patterns and preferences. Through comprehensive consideration of personal interests, POIs’ popularity and route constraints, the data and parameters calculated by the above preprocessing steps are used to obtain the results based on our improved HMM method. Based on the improved HMM method, the Viterbi algorithm is used to generate the visit sequence of POIs which is the optimal path. The route takes the POIs’ popularity, personal interests and distance as objective functions to calculate. Therefore, the recommended route with the best POIs will be the optimal route to meet the user’s needs according to the user’s personal interests.

### 4.2. Data Acquisition and Pre-Processing

Several different types of data are obtained from the crowd sourcing spatiotemporal data, including road network data, POIs data and POI heat data.

The road network data were obtained from the OSM (OpenStreetMap) platform, which is a road system consisting of various roads within a certain region. OSM is a free open source and editable map service which is created by the online public. In this paper, the road network data were collected in the main urban area of Changsha over the prior two years. There were 16,597 road segments in total, which formed the road network G in the main urban area of Changsha. The road segments will be defined as $r_{j}$. In other words, the road network is composed of road segment $G=(r_{1},r_{2},r_{3},\cdots ,r_{j})$. Each segment contains unique FID (factor ID) values, the start and end points of the road segment, the length of the road segment and the latitude and longitude of the road segment.

A POI is defined as a distinctive physical location in the real world (school, shopping mall, park, etc.). In this paper, POI data were mainly obtained from Baidu map and OpenStreetMap. There are 82 POIs in total. The basic attributes of each POI include the latitude and longitude information. These 82 POIs are divided into 15 different subjects according to the type of POI. $s_{i}$ denotes a subject and $p_{ij}$ denotes a POI. All POIs within each topic are denoted as $s_{i}=\left(p_{ij}|i=1,2,\cdots ,j\right)$, where $p_{ij}$= ($p_{ij}$ latitude, $p_{ij}$ longitude).

Heat data of POIs [47] is obtained from the big data display platform. Heat data of POIs refer to dissemination of Internet-based content and users’ interaction data, which means the popularity of POIs. The big data display platform based on massive user data can quickly obtain heat trends through people’s searches for a keyword on PCs or portable terminal. That is done in order to understand the real needs of users and understand the crowd attributes of keyword search. In our research, the monthly attention index data of each POI in 2018 were mainly obtained as the heat values of POIs.

Due to the complexity of the road network, we must make sure of the correctness of the road network. Before the route planning, detecting the correctness of the road network is a necessary step. The wrong road network data should be modified and deleted. Further, some abnormal POIs have to be deleted and modified.t The POIs data which are required will be left at the end. The location of the POI will not appear on the road. Therefore, it is necessary to calculate the projected point of each POI on the corresponding road segment. The shortest route between candidate points is found through network analysis tools, and the path distance is obtained. The distance of the great circle is calculated by two POIs. The above is the pre-processing step.

### 4.3. HMM for Route Planning

**Figure 3:**
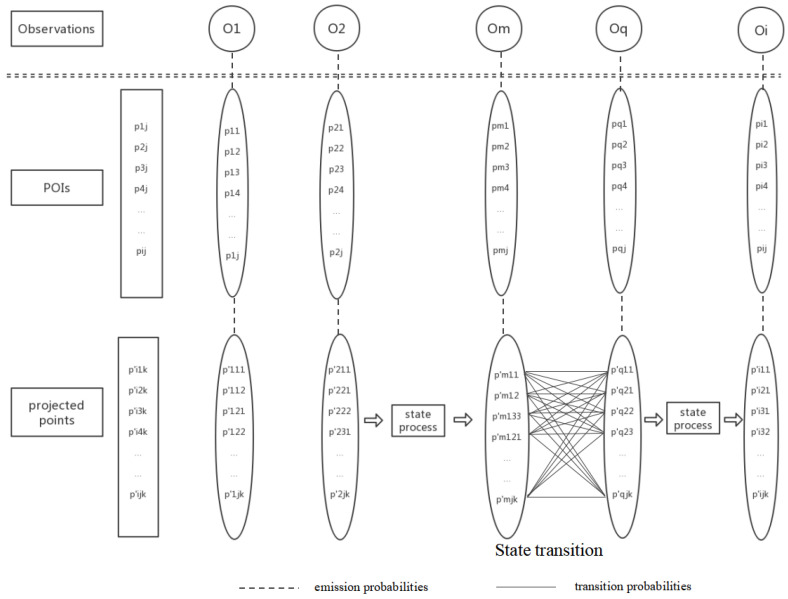
The improved HMM structure for route planning.

The object of a HMM is to model the state sequence over time. The state space for a HMM model includes a set of observed states; each of them is associated with an emission probability and a chain of hidden states made up of hidden variables. They are the basic components of the HMM. The improved HMM-based route planning of personal preferences models the route planning process with the Markov process. Figure 3 shows the improved HMM model. Unlike the previous state space composition, the improved HMM model consists of three layers of state space. Firstly, it is the state $O_{n}$ that the user can observe, which is represented by the category of POI. The part above the double dotted line indicates the observed state. Multiple observation states selected by the user constitute an observation sequence. Then, the hidden state space is composed of two hidden layers: The first hidden layer is composed of all candidate POIs $c_{i}$ in each observed state. All projected points $c_{ij}$ form the second hidden layer; the transition probability between the hidden states occurs in this layer. The part below the double dotted line indicates the hidden state. In addition, the emission probability is represented by a dotted line. The transition probability is represented by a solid line. These layers reflect the state vectors of all possible states.

The improved HMM model is described as follows:

#### 4.3.1. Hidden States

Hidden states are actually hidden in the Markov model and satisfy the Markov property. In this paper, the hidden state space is composed of two hidden layers: The first hidden layer which consists of all candidate POIs $p_{ij}$ in each observed state, is a set $\left(p_{i1},p_{i2},p_{i3},\cdots ,p_{ij}\right)$; The second hidden layer which consists of all projected points $p_{ijk}^{\prime }$, is a set $\left(p_{ij1}^{\prime },p_{ij2}^{\prime },\cdots ,p_{ijk}^{\prime }\right)$.

#### 4.3.2. Observations

Observations are obtained by direct observation and associated with the hidden state in the model. In this paper, it is the observed state $O_{n}$ that the user can observe, which is represented as a theme of POI. Multiple observation states selected by the user constitute an observation sequence $\left(o_{1},o_{2},o_{3},\cdots ,o_{n}\right)$.

#### 4.3.3. Emission Probabilities

The emission probability indicates the emission probability distribution in the state of the system. It is expressed as the calculated probability value under the condition of hidden state $p_{ijk}^{\prime }$ and at time t. In this method, the emission probability is calculated by the heat value of the POI, which means calculating the attention values of all POIs for a subject at different times and their projections to the road segment. The Euclidean distances between POIs and their corresponding points of projection are calculated. By multiplying the two values, the emission probability of each POI is obtained. Based on previous work [40], the formula of the emission probability is defined $pr(p|p_{ijk}^{\prime })$ as:

(2) $$p\left(p_{ij}\right)=\frac{Hp_{ij}}{\sum_{i=1}^{n}Hp_{ij}}$$

(3) $$pr\left(p|p_{ijk}^{\prime }\right)=p\left(p_{ij}\right)\frac{1}{\sqrt{2\pi }\delta_{c}}e^{-0.5{\left(\frac{∥p_{ij}-p_{ijk}^{\prime }∥Euclideandistance}{\delta_{c}}\right)}^{2}}$$

#### 4.3.4. Transition Probabilities

**Figure 4:**
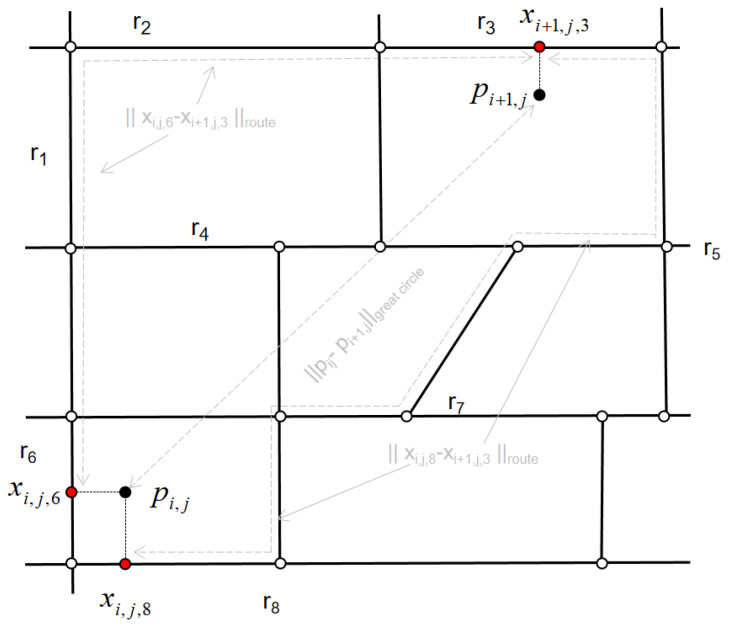
This shows the projected points of two points-of-interest (POIs) on the road network. Calculate the great circular distance between POIs. Calculate the path distance between projected points.

The state transition probability shows the probability when the state transitions from one state to another state. In this paper, transition probabilities give the probability of movement between the candidate points at the two times. Due to the uncertainty of the first POI, there is no need to calculate the initial state probability. Specifically, for observed points $p_{ij}$ and projected points $p_{ijk}^{\prime }$, the latitude/longitude of the projected point are $x_{i,j,k}$. For the next observation point $p_{i+1,j}$ and the projected point $p_{i+1,j,k}^{\prime }$, the latitude/longitude of the projected point are $x_{i+1,j,k}$. After determining the location of the projected point, the distance is calculated. We refer to this distance as the "route distance," noted as $∥x_{i,j,k}-x_{i+1,j,k}∥$. The great circle distance between these two POIs is $∥p_{ij}-p_{i+1,j}∥$. Figure 4 shows an example of a distance calculation. The algorithm describes the projected points of two POIs on the road network. It calculates the great circle distances between POIs and the path distances between projected points. The formula of the transition probability is defined p(dt) as:

(4) $$p\left(dt\right)=\frac{1}{\gamma }e^{-dt/\gamma }$$

(5) $$dt=\frac{\left|{∥p_{ij}-p_{i+1,j}∥}_{greatcircle}-{∥x_{i,j,k}-x_{i+1,j,k}∥}_{route}\right|}{timeinterval*timeinterval}$$

γ is a probability parameter that describes the difference between route distances and great circle distances. The time interval is the speed of the vehicle; it is obtained by the ratio of the route distance between two projected points to the speed limit of the road. In this paper, the speed limit of the road refers to the speed limit standard of urban road vehicles in the study area. The calculation of the parameters will get a detailed explanation in the experimental section.

### 4.4. Optimal Path

The emission probabilities of each candidate POI and the transition probabilities between the candidate projected points are calculated by the above equation. The Viterbi algorithm through the HMM is used to obtain the optimal route and the projected points which constitute the route. The Viterbi algorithm is a dynamic programming algorithm. It is used to find the Viterbi path that is most likely to produce a sequence of observed events. The algorithm multiplies the emission probability and the transition probability to maximize the probability. The final result is the route with the highest probability, that is, the optimal route.

Algorithm 1 describes the workflow of an improved HMM-based approach for planning individual routes. It takes the set of observation states O, a sequence of observations S, POI dataset P, road network G and the recommendation of POI-PP (projected point) as inputs. The recommendation information of POIs is used first for calculating the popularity index of each POI. Then, the first layer of hidden state and the second layer of hidden state are constructed successively. In the first hidden state, it will check all the observation states corresponding to the sequence of observation and check all POIs in each observation state. The popularity index of each POI in the corresponding state is obtained. In the second layer of hidden state, it gets the projection of POI on the road segment in the second hidden state. These projections of POIs are represented as candidate points in each hidden state. In this algorithm, it takes each hidden state in $s_{i}$ to calculate parameters. The road network G is used to calculate the great circle distance GD between POIs, the route distance RD between projected points in different observation states and the great circle distance from POI to the projection PD. Next, the emission probability EP based on the popularity index and the distance PD are calculated. For each projection of the POI, it obtains the transition probability from $s_{i}$ to $s_{i_{1}}$. Finally, the EP and TP are imported into HMM and the following Viterbi algorithm us used to calculate the optimal route.
**Algorithm 1** An improved HMM-based approach for planning individual routes.**Input:** States of observation *O*; A sequence of observation *S*; POIs Dataset *P*; Road network *G*; The recommendation of POIs PP **Output:** Optimal personalized route *R*
1:Pop = calPopularityIndexInfo(P,PP);// Calculate the popularity index of each POI2:$S_{firstHidden}$ = Sequence(O,S,P,Pop); // All POIs in each observation state3:$S_{secondHidden}$ = Project($S_{firstHidden}$, *G*);// Get the projection of POI on the road segment4:**for** each hidden state in $s_{i}$ **do**5:    RD = getRouteDistance($S_{secondHidden}$, *G*);// RD represents the route distance between two projected points6:    GD = getGreatCircleDistance($S_{firstHidden}$, *G*);// GD represents the great circle distance between two POIs7:    PD = getPOIToProjectionDistance(*P*, $S_{secondHidden}$);// PD represents the great circle distance from POI to the projection8:    EP = getEmissionProbability($S_{secondHidden}$, PD);// EP represents the emission probability of the projection of the POI9:    **for** eachprojectionofthePOI **do**10:        TP= getTransitionProbability($S_{secondHidden}$, RD, GD);// TP represents the transition probability from $s_{i}$ to $s_{i-1}$11:    **end for**12:**end for**13:*R* = createIndividualRoutesModel(EP,TP,G);14:**return***R*;


## 5. Experiments

A series of experiments were conducted to evaluate the performance of the proposed method. To validate the proposed method, the real data were used for doing experiments in this section. First of all, we present the experimental data and settings, including the data and parameters which were used. Then, we report the results and compare them with those of other methods.

### 5.1. Experimental Data and Settings

In this paper, Changsha as the research area was selected. It is the capital of Hunan province, China, located in the east by north of Hunan, the lower reaches of the Xiangjiang river and the western edge of the Changliu basin. Changsha has jurisdiction over the six districts and administers two county-level cities. In this paper, we focus on five districts in the main urban area of Changsha, including Furong, Yuhua, Tianxin, Kaifu and Yuelu. Changsha is an important central city in the south of the Yangtze River in China, and it is one of the national comprehensive transportation hubs. In recent years, the economic development of Changsha has been in a leading position among the new first-tier cities. Therefore, it is necessary to take Changsha as the research area.

The road network data have been collected in the main urban area of Changsha in the past two years. There are 82 POIs which were selected as the experimental points. These 82 POIs were selected based on the ranking of the heat index of each subject. In addition, because these POIs have a wide distribution, they were divided into 14 different subjects according to the type of POI—university, shopping mall, historic site, theater, etc. Finally, the sequence of subjects was formed according to the actual requirements of the user to determine the best route. The following Table 1 is a brief description of the data obtained.

### 5.2. Parameter Estimation

In this paper, the improved HMM needs three probability-related parameters. One is $\delta_{c}$ [40], which is related to calculating the emission probability. Then, the Euclidean distances from all projected points to POIs are calculated. The MAD (median absolute deviation) is used to estimate the value of $\delta_{c}$:

(6) $$\delta_{c}=1.4826*mediant_{t}\left({∥p_{ij}-x_{i,j,k^{*}}∥}_{Euclideandistance}\right)$$

According to our experimental data, this value was $\delta_{c}=143.62$ m.

The other two parameters are related to the transition probability—γ [40] and timeinterval separately. The γ describes the difference between route distance and great circle distance, which is also calculated using the MAD. Timeinterval represents the time interval between two points. It is calculated by the ratio of the allowable driving speed of the route to the distance between two points.

(7) $$\gamma =\frac{1}{\ln2}median_{t}\left|{∥p_{ij}-p_{i+1,j}∥}_{greatcircle}-{∥x_{i,j,k^{*}}-x_{i+1,j,k^{*}}∥}_{route}\right|$$

(8) $$timeinterval=\frac{{∥x_{i,j,k}-x_{i+1,j,k}∥}_{route}}{velocity}$$

As the experimental objects changed in each experiment, the calculated value of γ changed. The specific value is related to the experimental data. To calculate the time interval, the speed was set to 40 km/h according to the road speed of urban roads in Hunan Province and the actual extracted road network data.

### 5.3. Experimental Results

#### 5.3.1. The Influence of Open Spatial Data on Route Planning

To test personal willing route planning based on HMM, in this study, five experiments were carried out to verify it. In each experiment, several subjects were randomly selected as the experimental object, and the best route was obtained by this method. Meanwhile, to verify the influences of different degrees of attention on the improved HMM for route planning, the degrees of attention data of different months and quarters were obtained to conduct experiments separately. For each experiment, the transition probability of each experiment is constant. The emission probability will vary with the degrees of attention data.

**Figure 5:**
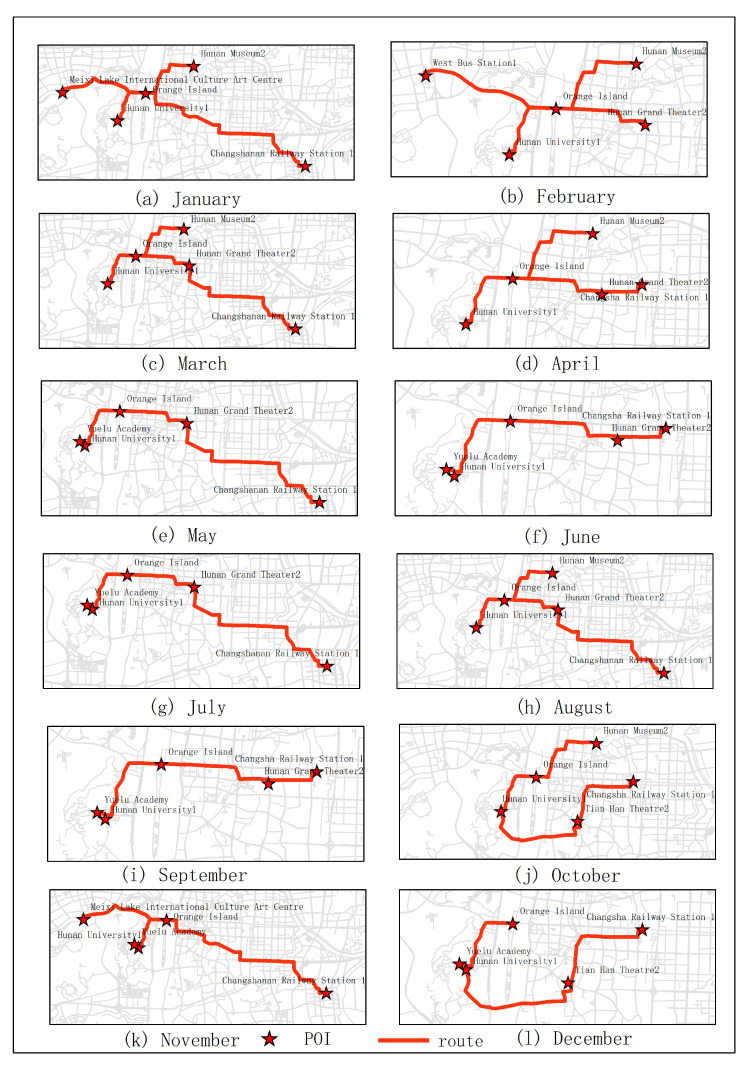
In experiment 1, five subjects were randomly selected. According to the popularity of each month, the optimal route was obtained through the improved HMM method.

In the first experiments, five subjects were selected for testing. They were city parks, museums, universities, theaters and stations. As shown in Figure 5. From the experimental results, it can be seen that the optimal route obtained in January was Juzizhou, Hunan Museum 2, Hunan University 1, Meixihu Theater and Changshanan Railway Station 1 (the number is expressed as the number of the projection point of the POI on the road segment). The optimal route obtained in June was Juzizhou, Yuelu Academy, Hunan University 1, Hunan Theater, Changsha Railway Station 1. The optimal route obtained in December was Juzizhou, Yuelu academy, Hunan University 1, Tianhan Theater, Changsha Railway Station. The optimal route obtained in the second quarter (June, July, August) was Juzizhou, Yuelu Academy, Hunan University 1, Hunan Theater, Changshanan Railway Station 1. The optimal route obtained in the fourth quarter (December, January, February) was Juzizhou, Hunan Museum 2, Hunan University 1, Meixihu Theater, Changsha Railway Station 1.

**Figure 6:**
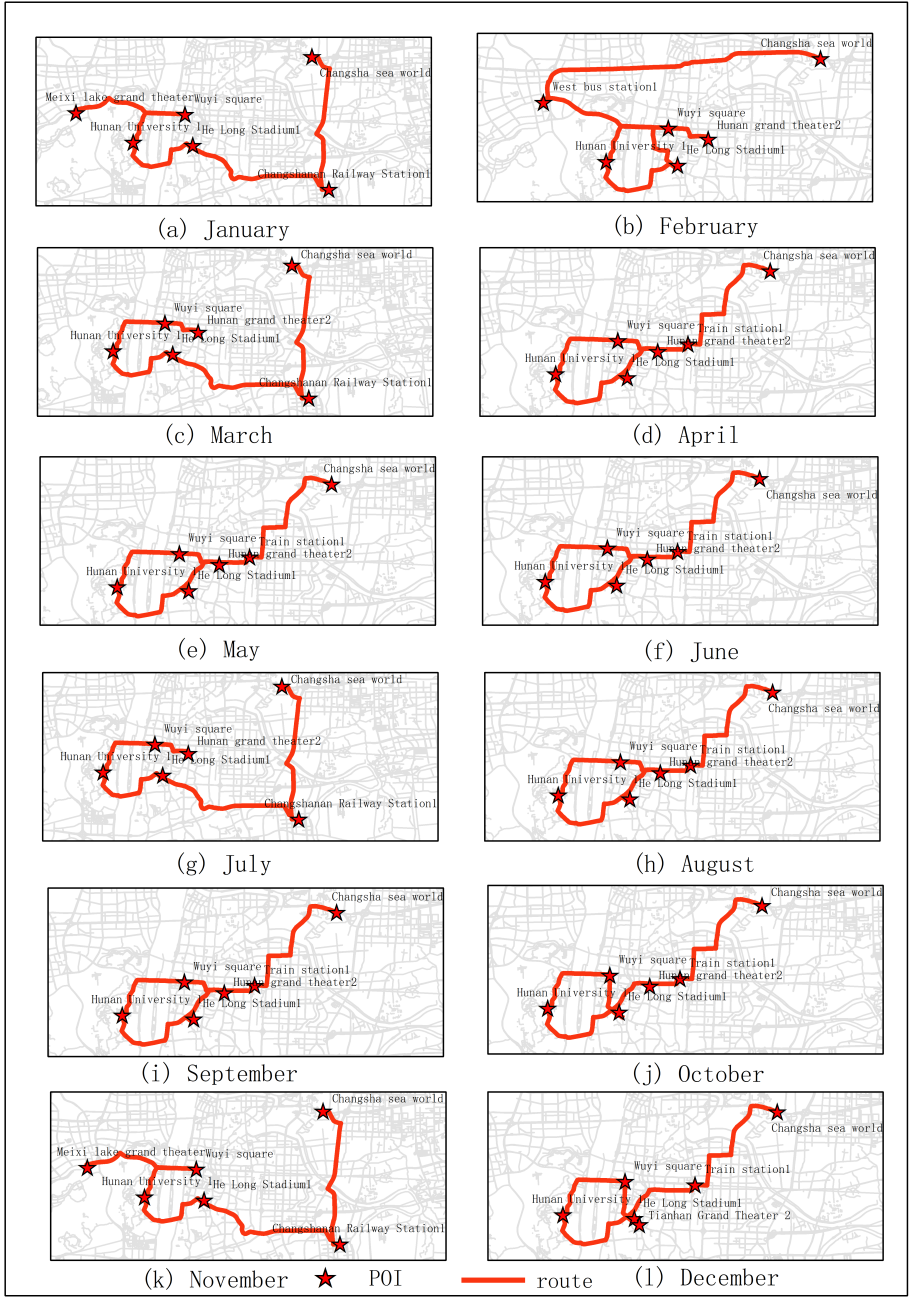
In experiment 2, six subjects were randomly selected. According to the popularity of each month, the optimal route was obtained through the improved HMM method.

**Figure 7:**
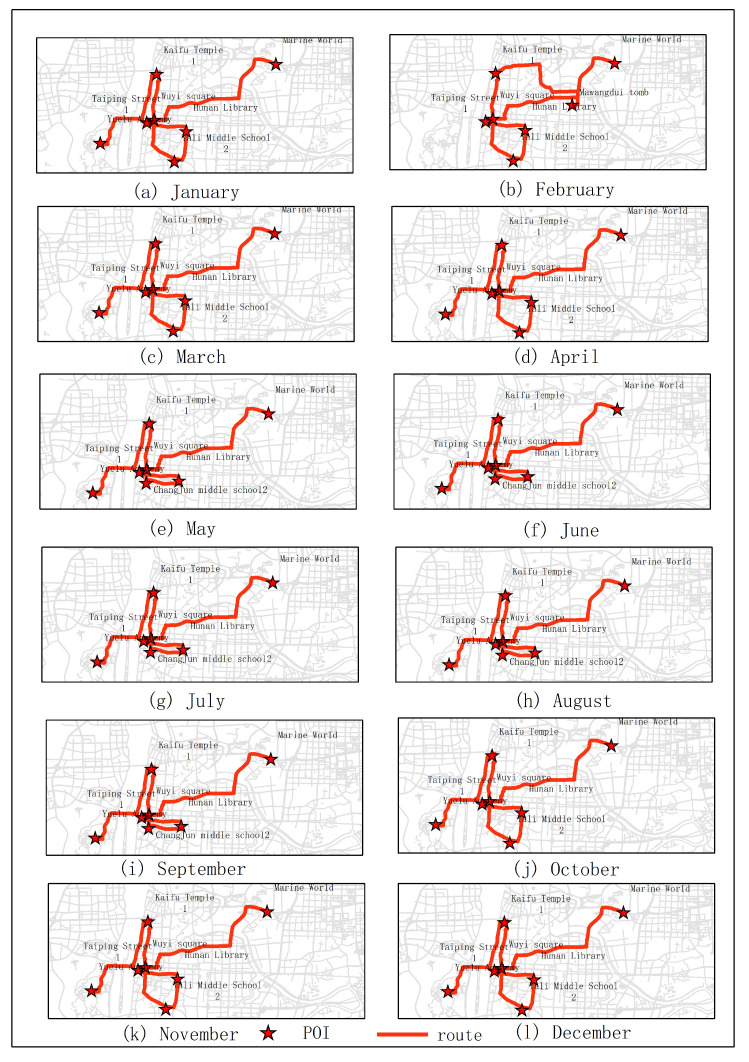
In experiment 3, seven subjects were randomly selected. According to the popularity of each month, the optimal route was obtained through the improved HMM method.

**Figure 8:**
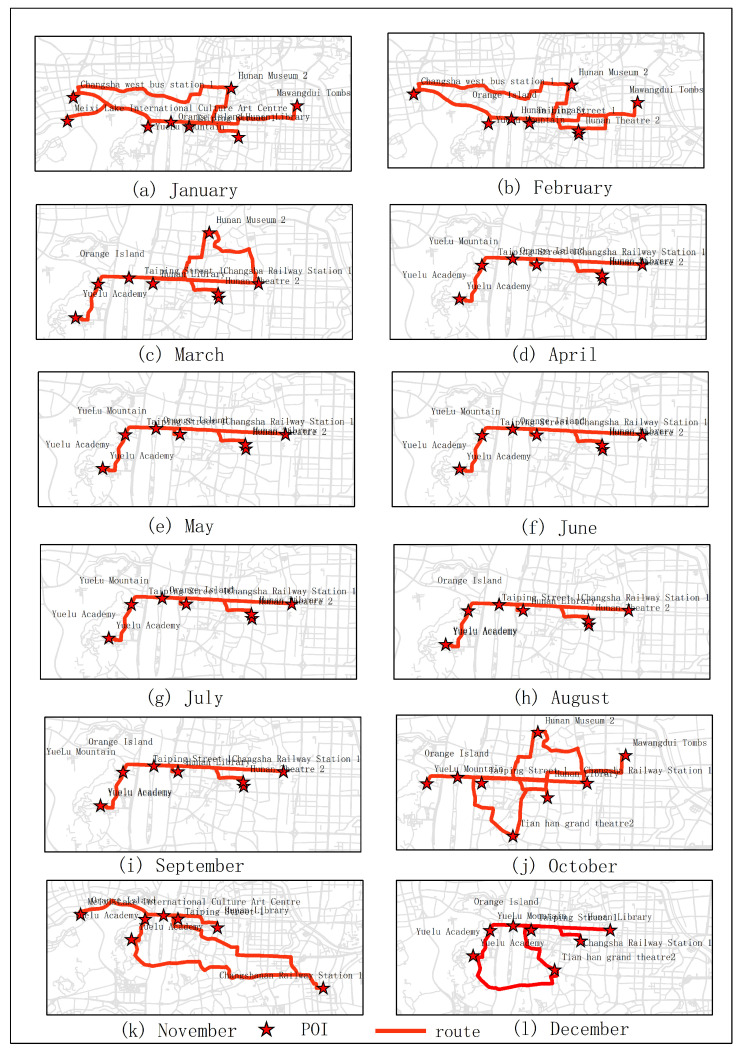
In experiment 4, eight subjects were randomly selected. According to the popularity of each month, the optimal route was obtained through the improved HMM method.

**Figure 9:**
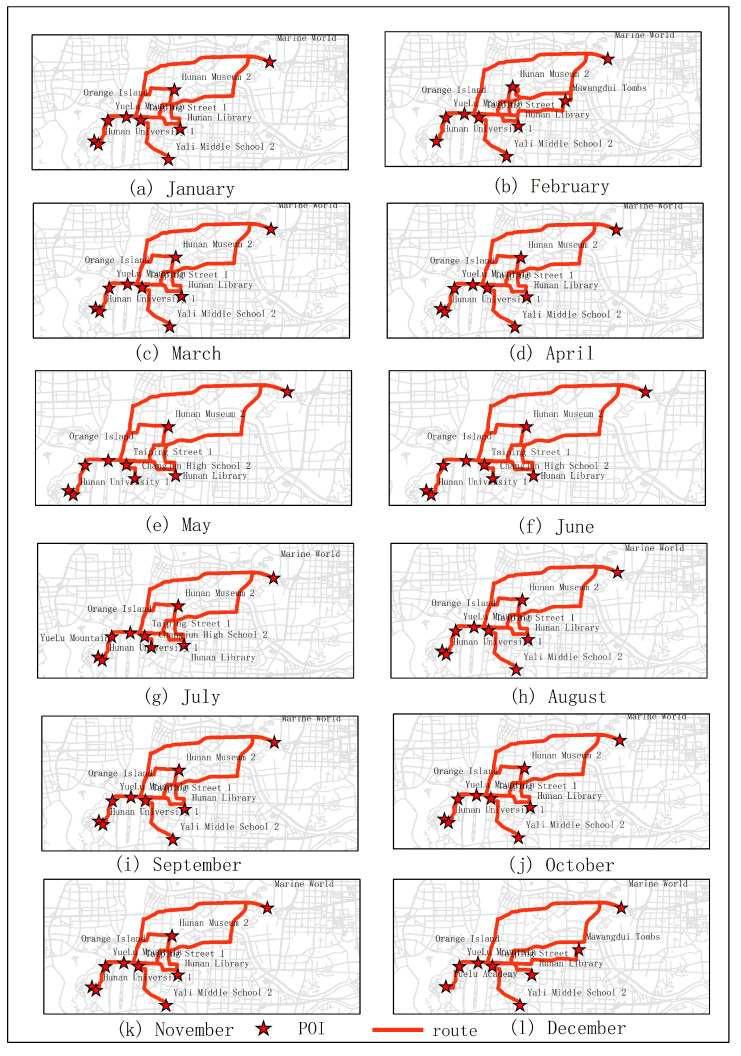
In experiment 5, nine subjects were randomly selected. According to the popularity of each month, the optimal route was obtained through the improved HMM method.

**Figure 10:**
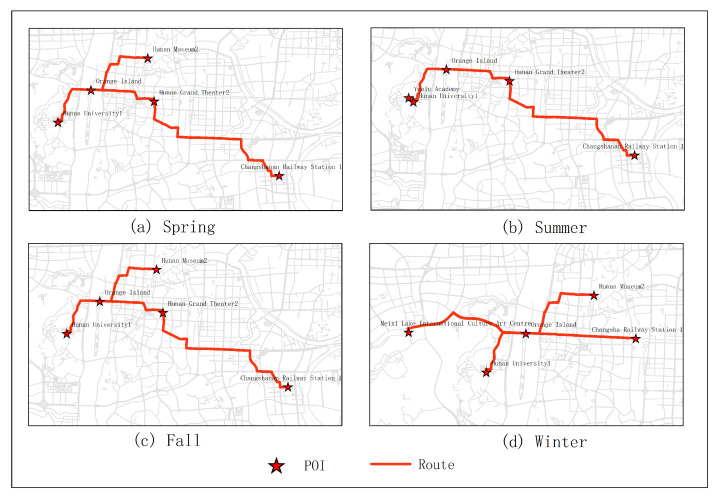
In experiment 1, five subjects were randomly selected. According to the popularity of each season, the optimal route was obtained through the improved HMM method.

**Figure 11:**
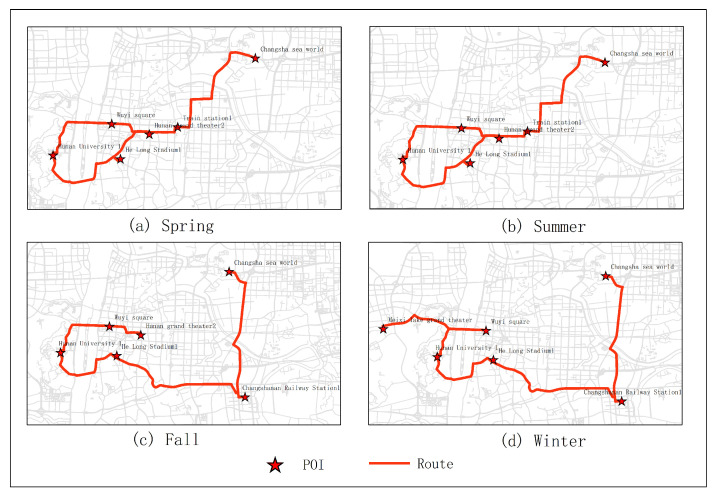
In experiment 2, six subjects were randomly selected. According to the popularity of each season, the optimal route was obtained through the improved HMM method.

**Figure 12:**
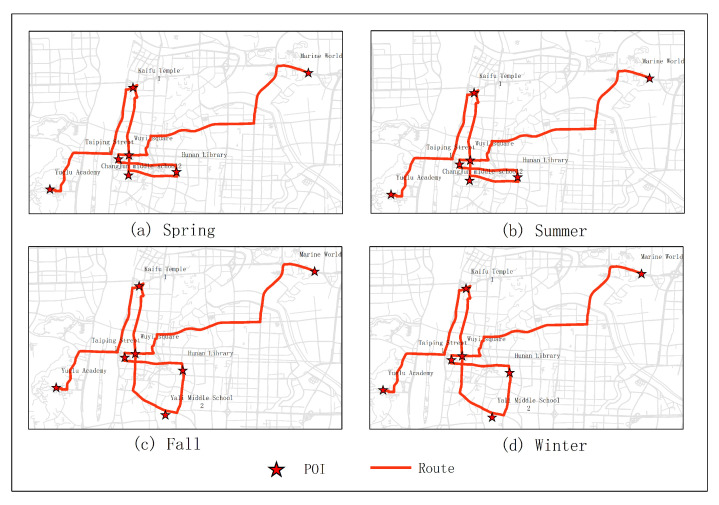
In experiment 3, seven subjects were randomly selected. According to the popularity of each season, the optimal route was obtained through the improved HMM method.

**Figure 13:**
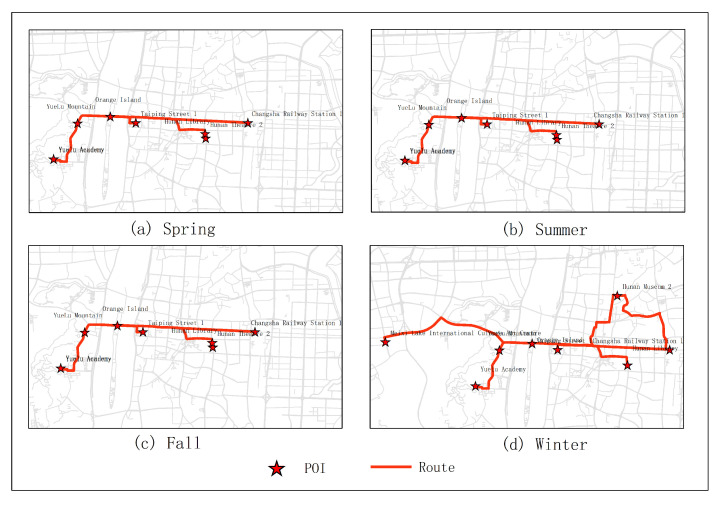
In experiment 4, eight subjects were randomly selected. According to the popularity of each season, the optimal route was obtained through the improved HMM method.

**Figure 14:**
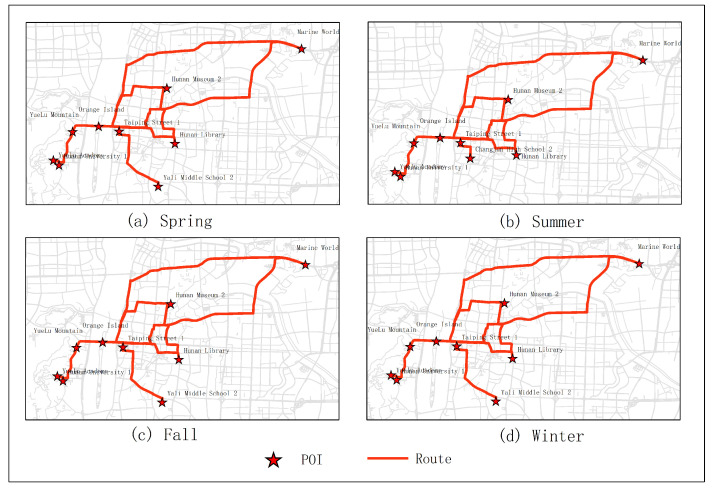
In experiment 5, nine subjects were randomly selected. According to the popularity of each season, the optimal route was obtained through the improved HMM method.

The results of optimal routes obtained by other experiments are shown in Figure 6, Figure 7, Figure 8, Figure 9, Figure 10, Figure 11, Figure 12, Figure 13 and Figure 14. From the experimental results, it can be seen that with the differences of months and quarters, the projected points of certain topics in the route will change accordingly. The reason for this change is that in different months and quarters, people’s attention and search volume for POIs are different. For example, a POI may not get as much attention in one month, but it may generate far greater interest in another month. With the increase of attention, the rate of selection of route planning will be increased. The POI with the highest attention does not mean that it is the best choice for this topic. At the same time, it will consider the length of the route distances between POIs and other factors. The best route planning is calculated. It can be seen that most of the POI candidate points are consistent with the highest value of attention for a POI in the topic. This fully shows that the degree of attention as an influential factor in the improved HMM model has a great relationship with the choice of POIs. It also shows that it is necessary to use the attention for POIs as a variable. In most route planning, the route planning service usually needs online data for working. In this way, it can make the suitable and best routes for people through their preferences. Considering the heat values of POIs in different months and quarters, this method reflects the changes of route with time and reflects the timeliness.

To test the efficiency of the improved HMM in route planning, the running time of each test was checked. The results show that the method has high efficiency, and the running time is fast. With the increase in the number of experimental targets, the running time increased slightly.

#### 5.3.2. Our Methods versus Base Methods

In this section, the proposed approach is contrasted with the original HMM, user questionnaire and Dijkstra. For the original HMM, the crowd sourcing of spatiotemporal data is no longer considered, but the route planning is still based on the original HMM algorithm. For the user questionnaire, a questionnaire is used to encourage everyone to select POIs. At the end of the day the POI with the highest proportion constitutes the optimal path. A total of 82 valid questionnaires were used for review in this article. For the Dijkstra algorithm, the shortest path is determined from one vertex to the remaining vertex, which solves the shortest path problem in the power graph.

**Figure 15:**
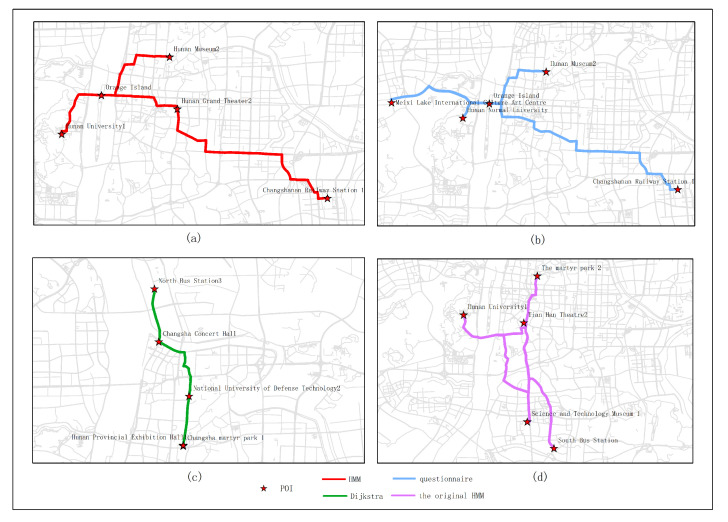
In experiment 1, five subjects were randomly selected, and the optimal route obtained by all methods is based on the data in March; among them, (**a**) shows the optimal route obtained by the improved HMM; (**b**) shows the optimal route obtained from the user questionnaire; (**c**) shows the optimal route obtained by Dijkstra; (**d**) shows the optimal route obtained by the original HMM.

**Figure 16:**
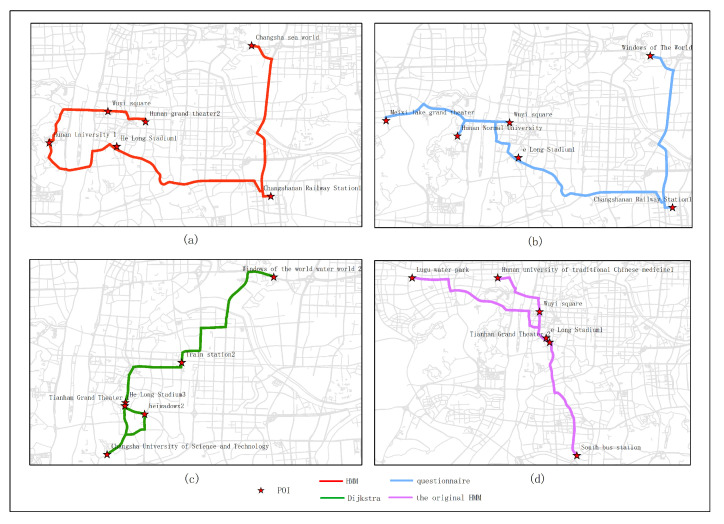
In experiment 2, six subjects were randomly selected, and the optimal route obtained by all methods is based on the data in March; among them, (**a**) shows the optimal route obtained by the improved HMM; (**b**) shows the optimal route obtained from the user questionnaire; (**c**) shows the optimal route obtained by Dijkstra; (**d**) shows the optimal route obtained by the original HMM.

**Figure 17:**
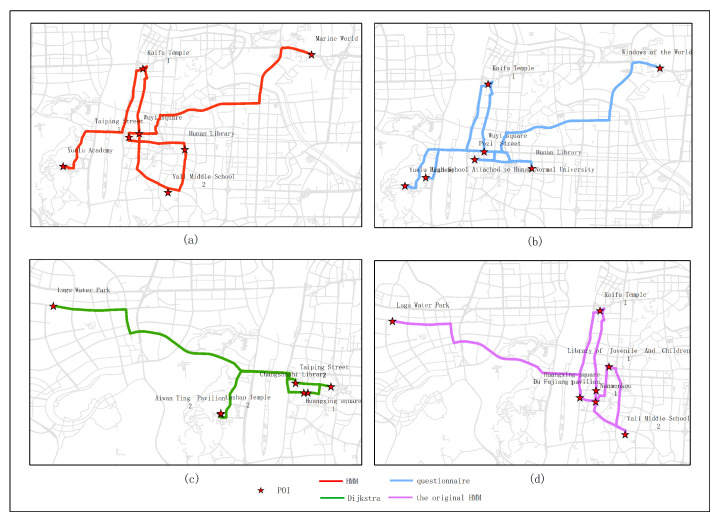
In experiment 3, seven subjects were randomly selected, and the optimal route obtained by all methods is based on the data in March; among them, (**a**) shows the optimal route obtained by the improved HMM; (**b**) shows the optimal route obtained from the user questionnaire; (**c**) shows the optimal route obtained by Dijkstra; (**d**) shows the optimal route obtained by the original HMM.

**Figure 18:**
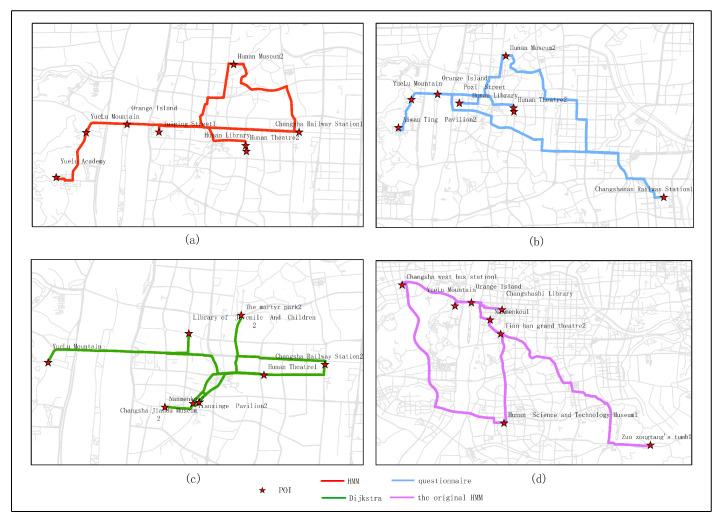
In experiment 4, eight subjects were randomly selected, and the optimal route obtained by all methods is based on the data in March; among them, (**a**) shows the optimal route obtained by the improved HMM; (**b**) shows the optimal route obtained from the user questionnaire; (**c**) shows the optimal route obtained by Dijkstra; (**d**) shows the optimal route obtained by the original HMM.

**Figure 19:**
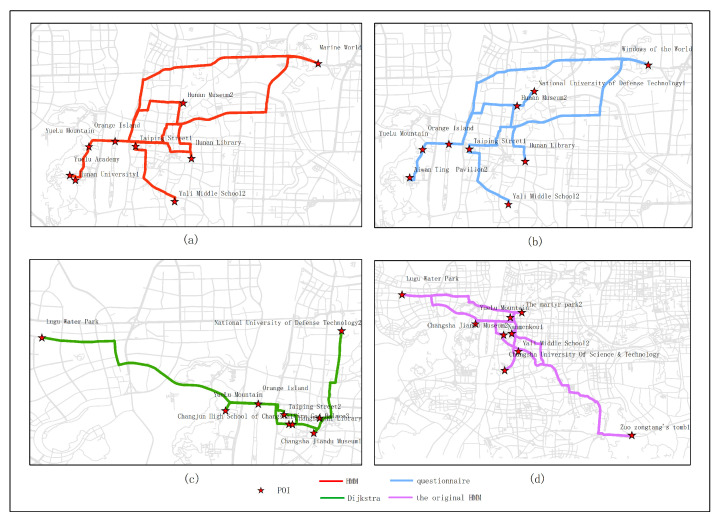
In experiment 5, nine subjects were randomly selected, and the optimal route obtained by all methods is based on the data in March; among them, (**a**) shows the optimal route obtained by the improved HMM; (**b**) shows the optimal route obtained from the user questionnaire; (**c**) shows the optimal route obtained by Dijkstra; (**d**) shows the optimal route obtained by the original HMM.

Figure 15, Figure 16, Figure 17, Figure 18 and Figure 19 show the optimal routes obtained by all methods; among them, (a) shows the optimal route obtained by the improved HMM; (b) shows the optimal route obtained from the user questionnaire; (c) shows the optimal route obtained by Dijkstra’s algorithm; (d) shows the optimal route obtained by the original HMM.

Different routes were obtained by different methods. The original HMM does not consider the heat value of POI; it can be seen from the above road map that the choice of POIs is more dependent on the determination of the previous POI, and there is no basis for the obtained route. It can be seen that the Dijkstra method and the original HMM only consider the distance factor while ignoring other factors that affect route selection. The result of the route may be a relatively unpopular route composed of POIs with low heat value, which is not attractive to users. The results of the questionnaire survey are compared with the results calculated by the method in this article. In a certain period, comparing the data obtained from offline and online, people’s choices of POIs are roughly similar. However, when acquiring offline data, people’s choices may be affected by the surrounding environment or these data are not as much as online data. It will lead to some discrepancies in the choice of POIs. The figure shows that the coincidence proportion of routes is relatively large, which proves the validity of our method. It implies that improved HMM plays an important role when performing successive personalized route planning. It can provide users with an optimal route that satisfies users’ needs, the distance and the popularity of POIs in a short period.

**Figure 20:**
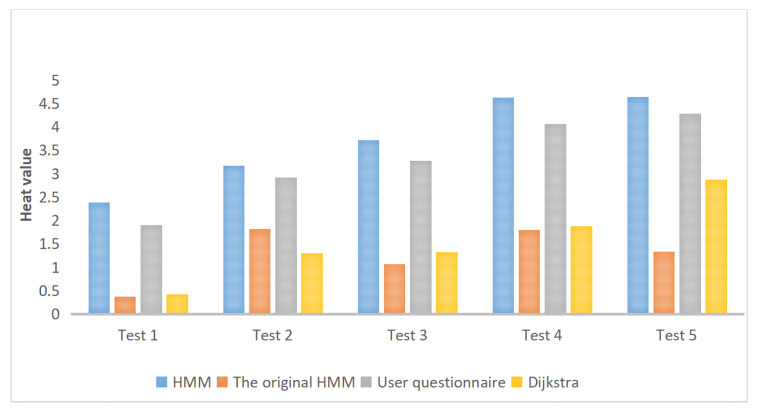
Changes of heat values for POIs in different experiments of the four methods.

Table 2, Table 3, Table 4, Table 5 and Table 6 are based on the route results from user surveys (because the survey was completed in March, the heat values of POIs in our route were calculated based on the data in March). We calculated the similarities between POIs, the similarities between the routes and the heat values of POIs of all methods. The similarities between POIs refer to the coincidence ratios between the POIs recommended by different methods and the POIs obtained from user survey route results in each experimental route. The similarity between the routes refers to the coincidence ratio between the route planning by different methods and the survey in each experimental route. The heat value of POIs refers to the total heat value of all POIs recommended by different methods in a certain month. The results show that the method proposed in this paper has a relatively high proportion compared with other methods. Both the POI similarity and the route similarity are better than those of the other two methods, and the heat value of POI is the highest (Figure 20). It also shows that the route obtained by our method is more in line with the needs of users and more close to the actual ideas of the user.

The method suggested in the paper could provide an optimal route for the user. Second, it recognizes the user’s individual needs and desires. The best route can be designed according to the points of interest chosen by the users. The proposed route incorporates thermal details from the normal POI. On the one hand, users need to find a lot of details while planning their own routes, which can take a long time. On the other hand, users can neglect those factors that need to be considered. This approach saves a lot of time compared to users looking for a mass of information on the Internet and preparing their routes. In the meantime, a range of variables are considered to provide suitable routes for users.

## 6. Conclusions

The importance of crowd sourcing spatiotemporal data from different LBSs was realized after a significant amount of research on route planning. In order to hit time-sensitive and customized paths, people’s willingness and spatial-temporal variables should be dynamically combined. This calls for new methods of route planning that can dynamically map public and individual desires into a realistic geographical environment.

In this paper, an improved dual-layer HMM was explored in the direction of associating one’s interests with crowd sourcing of spatiotemporal data, e.g., public preferences at different times, POIs and the road network, in order to generate personal routes. Based on the improved HMM, the proposed approach is versatile for the planning of personalized routes within urban road networks. The contributions of this paper are: (1) to reduce the impact of spatial uncertainty generated by conventional approaches on POIs and road network data; (2) to incorporate public preferences (from crowd sourcing to spatiotemporal data) and customized trends; (3) to map dynamic individual priorities in crowd sourcing spatiotemporal data to physical road network space; and (4) to emphasize the spatiotemporal dynamics, that is, the interactions between POIs and the environment in different periods.

If our future work can get more accurate and richer data, it will be improved in combination with other calculation models or methods to make the routes more refined. In terms of the spatiotemporal scale—analysis of the characteristics of the temporal and spatial distributions of the tourist flow—optimal route planning is constantly improved from the point of view of tourism choice. Taking into account the value and importance of the time budget, the evaluation criteria of the POI, the social relationship between POIs, etc., further research can be carried out so that tourists’ satisfaction with the routes can be improved.

## Figures and Tables

**Table 1:** Data description.

Data Type	Description	Feature	Data Source
Road network data	a road system consists of various roads within a certain region	contain 16,597 roads and each segment contains unique FID values, the start and end points of the road segment, the length of the road segment, and the coordinates of the road segment.	OSM
POI data	a distinctive physical location in the real world	contain 82 POIs in total. The basic attributes of each POI include the latitude and longitude information of POI and divided these 82 POIs into 15 different subjects according to the type of POI.	Baidu map and OSM
POI heat data	dissemination of Internet-based content, and users’ interaction data	the monthly and quarterly attention index data of each POI in 2018 was mainly obtained as the heat value of POI.	the big data display platform

**Table 2:** Comparison of methods in experiment 1.

Method	Number of Topics	Similarity—POIs	Similarity—Routes	Heat Value of POIs
Improved HMM	5	0.6000	0.4153	2.3907
Original HMM	5	0	0.0085	0.3734
User questionnaire	5	1	1	1.9002
Dijkstra	5	0	0	0.4159

**Table 3:** Comparison of methods in experiment 2.

Method	Number of Topics	Similarity—POIs	Similarity—Routes	Heat Value of POIs
Improved HMM	6	0.5000	0.6338	3.1723
Original HMM	6	0.3333	0.2083	1.8176
User questionnaire	6	1	1	2.9173
Dijkstra	6	0	0.0267	1.2976

**Table 4:** Comparison of methods in experiment 3.

Method	Number of Topics	Similarity—POIs	Similarity—Routes	Heat Value of POIs
Improved HMM	7	0.5714	0.6541	3.7117
Original HMM	7	0.1429	0.1050	1.0709
User questionnaire	7	1	1	3.2734
Dijkstra	7	0.1429	0.1000	1.3270

**Table 5:** Comparison of methods in experiment 4.

Method	Number of Topics	Similarity—POIs	Similarity—Routes	Heat Value of POIs
Improved HMM	8	0.6250	0.2745	4.6351
Original HMM	8	0.2500	0.0460	1.7980
User questionnaire	8	1	1	4.0650
Dijkstra	8	0.1250	0.0962	1.8808

**Table 6:** Comparison of methods in experiment 5.

Method	Number of Topics	Similarity—POIs	Similarity—Routes	Heat Value of POIs
Improved HMM	9	0.6667	0.8014	4.6427
Original HMM	9	0.2222	0.1366	1.3296
User questionnaire	9	1	1	4.2838
Dijkstra	9	0.2222	0.0789	2.8712

## Abbreviations

The following abbreviations are used in this manuscript:

LBS	Location-Based Services
HMM	Hidden Markov Model
POI	Point-of-Interest
FPMC-LR	Personalized Markov Chains and the Localized Regions
LBSNs	Location Based Social Networks
STELLAR	Spatial-Temporal Latent Ranking
DRPS	Dynamic Recommendation of POI Sequence
MEAP-T	Time-Aware Metric Embedding Approach with Asymmetric Projection
SPR	Sentimental-Spatial POI Recommendation
LOD	Linked Open Data
HMTP	Hidden Markov model-based Trajectory Prediction
PPM	Prediction by Partial Matching
OSM	Open Street Map
FID	Factor ID
MAD	Median Absolute Deviation
